# Immunologic impact of maraviroc in clinical practice of a university hospital

**DOI:** 10.1186/1758-2652-13-S4-P39

**Published:** 2010-11-08

**Authors:** S Plata Paniagua, N Trovato Lopez, I Castillo Romera, A Ais Larisgoitia, J Bellon Cano, M Sanjurjo Saez

**Affiliations:** 1 Hospital general Universitario Gregorio Marañon, Pharmacy, Madrid, Spain; 2 Hospital general Universitario Gregorio Marañon, Preventive Medicine, Madrid, Spain

## Purpose of the study

Maraviroc (MVC) is one of newest antiretrovirals. Several studies have assessed the impact of MVC on CD4 count, although few have analyzed it in clinical practice. We assessed the immunological impact of MVC in clinical practice in a university hospital.

## Methods

Observational, retrospective, and cohort study in adult outpatients taking antiretroviral therapy. We selected two cohorts: the MVC cohort (MVCC), comprising patients who had switched to MVC between 01/05/2008 and 31/07/2009, and the last-generation antiretroviral cohort (LGAC), comprising patients who had switched to darunavir, etravirine, and/or raltegravir during the same period. The primary endpoint was the increase in CD4 count at 48 weeks.

## Results

The MVCC included 21 patients and the LGAC 56. Only 18 of the 21 patients were finally analyzed, as 3 patients stopped MVC and did not reach 48 weeks. There were no differences in age (median MVCC, 46.93 years; LGAC, 45.43 years) or sex (MVCC, 66.1% men; LGAC, 61.9% men) between the cohorts. HIV was multidrug-resistant (genotypic resistance to drugs of iÜ2 classic families and/or extensive experience with 3 classic families) in 73.2% of LGAC and 95.2% of MVCC (p=0.094). Adherence during the previous year of treatment with MVC was >90% in 85.7% of MVCC and 76.6% in LGAC. There were no significant differences between the cohorts. Raltegravir was included in the regimen in 86% of the MVCC and in 89% of the LGAC. At baseline, viral load was undetectable (defined as <50 copies/ml) in 19% of the MVCC and 51.8% of the LGAC (p=0.02). At the end of follow-up (48 weeks), viral load was undetectable in 90.5% of the MVCC and in 85.7% of the LGAC (p=0.86). At baseline, the mean CD4 count was 362.67 in the MVCC and 365.64 in the LGAC (p=0.96); at the end of follow-up, these values were 507.24 (95% CI, 377.40-637.08) and 462.07 (95% CI, 367.14-556.99) for MVCC and LGAC, respectively, with a mean difference of 45 cells (95% CI, "C126.80 to 217.14). The mean increase in CD4 count was 145 cells (95% CI, "C7.037 to 296.17) vs. 96 cells (95% CI, "C30.44 to 223.30)

## Conclusions

An increase in the immune response was observed in both cohorts at 48 weeks of follow-up. Although the increase in CD4 count was higher in the MVCC, significant differences were not found.

